# Association between Dyslipidemia and Mercury Exposure in Adults

**DOI:** 10.3390/ijerph18020775

**Published:** 2021-01-18

**Authors:** Purum Kang, Hye Young Shin, Ka Young Kim

**Affiliations:** 1 College of Nursing, Woosuk University, Wanju 55338, Korea; kpurum@gmail.com; 2 National Cancer Control Institute, National Cancer Center, Goyang 10408, Korea; hs103740@ncc.re.kr; 3 College of Nursing, Baekseok Culture University, Cheonan 31065, Korea; 4 Department of Nursing, College of Nursing, Gachon University, Incheon 21936, Korea

**Keywords:** dyslipidemia, mercury, Hg, exposure, heavy metals

## Abstract

Background—Dyslipidemia is one of the prominent risk factors for cardiovascular disease, which is the leading cause of death worldwide. Dyslipidemia has various causes, including metabolic capacity, genetic problems, physical inactivity, and dietary habits. This study aimed to determine the association between dyslipidemia and exposure to heavy metals in adults. Methods—Using data from the seventh Korean National Health and Nutrition Examination Survey (2016–2017), 5345 participants aged ≥20 years who were tested for heavy metal levels were analyzed in this study. Multiple logistic regression was conducted to assess the factors affecting the prevalence of dyslipidemia. Results—The risks of dyslipidemia among all and male participants with mercury (Hg) levels of ≥2.75 μg/L (corresponding to the Korean average level) were 1.273 and 1.699 times higher than in those with levels of <2.75 μg/L, respectively. The factors that significantly affected the dyslipidemia risk were age, household income, body mass index, and subjective health status in both males and females. Conclusions—In adult males, exposure to Hg at higher-than-average levels was positively associated with dyslipidemia. These results provide a basis for targeted prevention strategies for dyslipidemia using lifestyle guidelines for reducing Hg exposure and healthy behavioral interventions.

## 1. Introduction

The increasing popularity of Western diets in Korea is increasing the importance of dyslipidemia [1,2]. Dyslipidemia is one of the common risk factors and predictors for cardiovascular disease, which is associated with high mortality rates due to myocardial infarction or stroke [3,4,5]. In addition, abnormal cholesterol metabolism with dyslipidemia is a public health issue since it can aggravate the status of patients with chronic disease [6,7,8]. These characteristics make the aggressive management of dyslipidemia necessary. There are several risk factors for dyslipidemia, including metabolic capacity, genetic problems, physical inactivity, and dietary habits [9,10], and its progression is a complex process due to dietary nutrition and metabolism being influenced by genetic factors. Modifications to the metabolism of lipid proteins are also involved in dyslipidemia [11]. However, dyslipidemia is not always the consequence of lifestyle, aging, and genetic problems since it has recently been reported that dyslipidemia may be associated with exposure to various environmental hazard factors, including heavy metals.

Heavy metals, including cadmium (Cd), lead (Pb), and mercury (Hg), are prevalent toxic substances that accumulate easily and are widely distributed in the environment. Exposure to heavy metals is suspected to alter energy metabolism, including that of lipid proteins. A study involving a mouse model showed that chronic Cd exposure induced the alteration of lipid metabolism [12]. Reportedly, cadmium interferes with antioxidant activity in normal cells and affects the overall metabolism [13]. Furthermore, cross-sectional studies showed a significant relationship between metabolic diseases and exposure to Cd [14]. The accumulation of Hg induced metabolic inactivity, including dyslipidemia by oxidative stress in humans [15], and a significant interrelation between lipid contents and the blood Hg level in older people was demonstrated [16]. Lead ions can disrupt cell metabolism by replacing other ions such as Ca^2+^, Mg^2+^, Fe^2+^, and Na^+^ in the human body [17]. It has also been shown that higher levels of heavy metals, including Pb, are related to altered levels of total cholesterol in adolescents [18].

Despite the potential of a close association between dyslipidemia and heavy metals, the actual impact of this association tends to be underestimated due to difficulties in proving it. However, there has been increasing concern about the potential for this health-related problem because the accumulation of heavy metals is associated with various diseases. Thus, this study aimed to determine the association between dyslipidemia and exposure to heavy metals in Korean adults based on the Korean National Health and Nutrition Examination Survey (KNHANES).

## 2. Methods

### 2.1. Data Source and Participants

This study analyzed data obtained from the seventh KNHANES (2016–2017) conducted by the Korea Centers for Disease Control and Prevention. The KNHANES uses a complex, stratified, multistage, and probability cluster sampling design as a nationwide population-based survey of the health and nutritional status of Koreans. The overall response rate for the seven KNHANES was 76.6%. The study analyzed 5345 of the 16,277 total KNHANES respondents aged ≥20 years who were tested for heavy metals. The study was approved by the university’s Institutional Review Board (no. 1044396-202004-HR-089-01). Ethical issues regarding plagiarism, informed consent, misconduct, data fabrication and/or falsification, double publication and/or submission, and redundancy have been completely observed by the author.

### 2.2. Study Variables: Demographic Factors

The following demographic characteristics of the participants were analyzed: sex, age, marital status, education level, occupation, household income, and residential area. Marital status was defined as being or not being currently married. Education level was classified into elementary school or below, middle school, high school, and university or above. Occupation was categorized into five groups: white-collar (WC) worker, including managers, professionals, and office workers; pink-collar (PC) workers, including service and sales workers; blue-collar (BC) workers, including technicians, as well as device and machine operators; agribusiness and low-level (AL) workers, including skilled workers in agriculture and fisheries, and laborers; unemployed. Household income was divided into four quartiles. Residential area was categorized into urban areas (administrative divisions of a city) and rural areas (areas not classified as administrative divisions of a city).

### 2.3. Study Variables: Health-Related Factors

The health-related factors included health behavior factors, such as drinking status, smoking status, body mass index (BMI), and the prevalence of dyslipidemia, as well as mental health factors, such as the subjective health status and stress level. Smoking status was categorized into current smoker (current smoking) and nonsmoker (former smoker or never). The drinking status was classified based on alcohol consumption into current drinking and not drinking.

The BMI was calculated as the weight in kilograms divided by the square of the height in meters and was categorized into normal weight (18.5–25.0 kg/m^2^), overweight (25.0–29.9 kg/m^2^), obesity (≥30 kg/m^2^), and underweight (<18.5 kg/m^2^). The presence of dyslipidemia was defined based on having been diagnosed with dyslipidemia by a physician. The subjective health status was divided into five categories: very good, good, moderate, poor, and very poor. For the analysis, these responses were condensed into the three categories of good (combining very good and good), moderate, and poor (combining poor and very poor). Stress levels were grouped into low, moderate, and high (combining very high and high).

### 2.4. Study Variables: Heavy Metal Testing

Blood Pb and Cd were measured using graphite furnace atomic absorption spectrometry with a Zeeman background correction (AAnalyst 600, Perkin Elmer, Turku, Finland). The blood Hg level was measured using a gold amalgam collection method (DMA 80, Milestone, Bergamo, Italy). The blood Pb, Hg, and Cd levels were dichotomized based on the corresponding Korean average levels found in the third Korean National Environmental Health Survey (KoNEHS) (2015–2017) into the following groups: Pb, <1.60 and ≥1.60 μg/dL; Hg, <2.75 and ≥2.75 μg/L; Cd, <0.36 and ≥0.36 μg/L [19].

### 2.5. Statistical Analysis

Sampling weights were applied to the participants to avoid bias in the national estimates and thereby ensure that the sample was representative of the Korean population. The chi-square test was used to assess the relationships of dyslipidemia with demographic, health-related factors, and heavy metal exposure. Multiple logistic regression models were analyzed to identify the factors that significantly affected the prevalence of dyslipidemia separately in males and females.

Statistical significance in this study was defined as a *p*-value of <0.05. The complex sample design was taken into consideration for the data analysis, which was conducted using SPSS software (version 25.0, IBM Corporation, Armonk, NY, USA).

## 3. Results

### 3.1. General Characteristics of the Study Population

Table 1 lists the following general characteristics of the study population quantified as unweighted numbers and weighted percentages or weighted means: age, marital status, education level, occupation, household income, residential area, drinking status, smoking status, BMI, prevalence of dyslipidemia, subjective health status, stress level, and exposure to heavy metals (Pb, Hg, and Cd). The 5345 study participants comprised 2424 males and 2921 females with a mean age of 43.7 years. Married participants and those with an education level of university or above predominated among both the males and females. Regarding occupation, those who were unemployed predominated among the total sample and females, with white-collar workers predominating among males. The largest proportion of participants had a household income in the fourth quartile (highest level) and resided in urban areas. Current drinking, nonsmokers, normal BMI, and no dyslipidemia diagnosis also predominated, except for male smokers. Moderate levels predominated for subjective health status and stress level. Regarding heavy metal exposure, the average blood lead, mercury, and cadmium concentrations were 1.60 μg/dL, 2.75 μg/L, and 0.36 μg/L in the third Korean National Environmental Health Survey. In this study, the Pb level was mostly <1.60 μg/dL among all participants and females, and mostly ≥1.60 μg/dL among males; the Hg level was mostly ≥2.75 μg/L among all participants and males, and mostly <2.75 μg/L among females; the Cd level was mostly ≥0.36 μg/L among all participants, males, and females.

### 3.2. Weighted Prevalence of Dyslipidemia According to Sex

The characteristics of the weighted population according to the diagnosis of dyslipidemia and sex are presented in Table 2. Age, marital status, education, occupation, household income, drinking status, BMI, subjective health status, and exposure to Pb, Hg, and Cd differed significantly between the dyslipidemia and non-dyslipidemia groups among all participants. Among males, there were significant differences between these two groups in age, marital status, education, smoking status, BMI, subjective health status, and exposure to Pb, Hg, and Cd. Among females, there were significant intergroup differences in age, marital status, education, occupation, household income, drinking status, smoking status, BMI, subjective health status, and exposure to Pb, Hg, and Cd.

### 3.3. Factors Affecting Dyslipidemia Risk According to Sex

**Figure 1:**
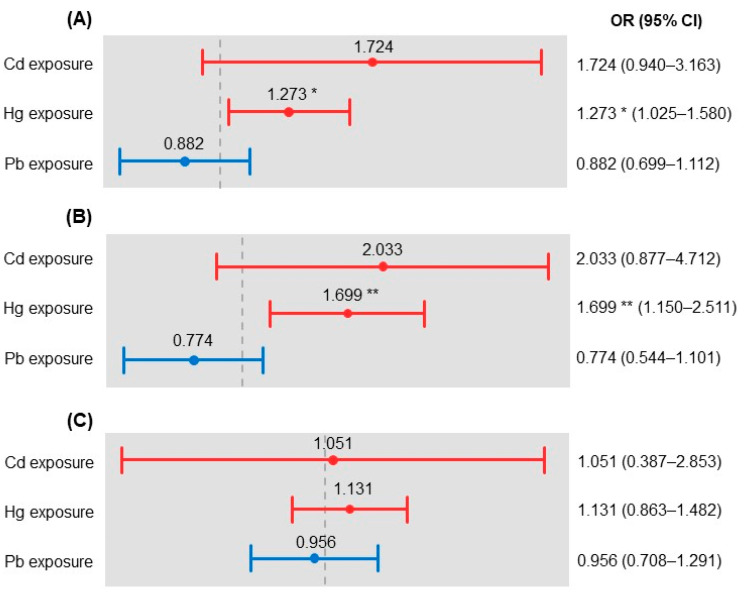
Heavy metals affecting dyslipidemia risk according to sex: (**A**) total sample, (**B**) male, and (**C**) female. The grey dotted lines represent OR = 1. * *p* < 0.05, ** *p* < 0.01.

The results of the multiple logistic regression analysis are presented in Table 3. The factors affecting the risk of dyslipidemia were age, household income, BMI, subjective health status, and Hg exposure among all participants. Among the total sample, the odds ratio (OR) for the dyslipidemia risk was 1.068 for age. Regarding household income, the risks of dyslipidemia were 1.571, 1.872, and 1.621 times higher for those in the second, third, and fourth quartiles, respectively, than for those in the first quartile. The ORs for the dyslipidemia risk were 1.558, 2.184, and 0.192 for those who were overweight, obese, and underweight, respectively, compared to those who were of normal weight. The ORs for the dyslipidemia risk were 1.821 and 3.115 for those with moderate and poor subjective health statuses, respectively, compared to those with a good status. Furthermore, the dyslipidemia risk was 1.273 times higher among those with Hg ≥2.75 μg/L than among those with Hg <2.75 μg/L (Figure 1A).

Among males, the significant factors affecting dyslipidemia risk were age, BMI, subjective health status, and Hg exposure. The OR for the dyslipidemia risk was 1.066 for age, and 1.423, 2.016, and 0.183 for those who were overweight, obese, and underweight, respectively, compared to those who were of normal weight. The OR for the dyslipidemia risk for someone with a poor subjective health status was 3.621 compared to a good status. Furthermore, the dyslipidemia risk was 1.699 times higher among those with Hg ≥2.75 μg/L than among those with Hg <2.75 μg/L (Figure 1B).

Among females, the significant factors affecting dyslipidemia risk were age, household income, BMI, and subjective health status. The OR for the dyslipidemia risk was 1.079 for age. Regarding household income, the risks of dyslipidemia were 1.980, 2.376, and 1.744 times higher for those in the second, third, and fourth quartiles, respectively, than for those in the first quartile. The ORs for the dyslipidemia risk were 1.567, 2.078, and 0.221 for those who were overweight, obese, and underweight, respectively, compared to those who were of normal weight. The ORs for the dyslipidemia risk were 2.141 and 2.665 for those with moderate and poor subjective health statuses, respectively, compared to those with a good status (Figure 1C).

## 4. Discussion

Heavy metals are non-biodegradable environmental chemicals that exert numerous adverse effects on humans. There is increasing interest in the adverse effects of exposure to heavy metals and its association with dyslipidemia. In this study, we focused on finding the association between exposure to heavy metals and dyslipidemia by analyzing multistage, stratified sampling data for adults. Our results showed that a higher Hg level in the blood was associated with dyslipidemia, with this association varying according to sex.

Mercury is one of the major heavy metals that exacerbate metabolic syndrome and cardiovascular disorders, including atherosclerosis [20,21]. A cross-sectional study found that the dysregulation of lipids was related to higher levels of Hg [22]. The present study showed that higher blood levels of Hg are significantly associated with dyslipidemia, which is consistent with the previous findings. Few previous studies have investigated the mechanisms underlying the effects of Hg in dyslipidemia. One possible mechanism involves the homeostasis of lipid metabolism and adipocytes [23]. Adipocytes are involved in lipid metabolism by producing adipokines [24], and Hg reportedly produced functional abnormalities in the adipose tissue of mice [25]. Furthermore, the toxic effects of Hg not only include oxidative stress but also the depletion of antioxidants [26]. Oxidative stress is a major factor that contributes to cell dysfunction and is linked to various diseases, including dyslipidemia [27]. Hg exposure induces the overproduction of reactive oxygen species following cell damage and the oxidation of low-density lipoprotein cholesterol [28]. Thus, it is speculated that pathogenesis is linked to abnormal lipid metabolism, which can develop into dyslipidemia.

A particularly interesting finding of the present study was that dividing heavy metal levels based on the Korean average level in the third KoNEHS revealed that the association of dyslipidemia with Hg exposure might be influenced by sex. There are several possible reasons for this result. Although there have been inconsistencies, some previous studies have found males to be more vulnerable to the adverse effects of Hg than females [29], which could be explained by sex differences in detoxification processes, including the oxidative stress pathway and the availability of glutathione. The primary route for eliminating Hg from the body is excretion in bile after binding to glutathione [30]. The plasma level of glutathione peroxidase has been reported to be higher in females than in males; therefore, Hg might exert greater effects in males [31]. An observational study found an association between altered lipid profiles and blood Hg in Korean male adolescents [32], which is consistent with our findings. Another possible reason is that males are exposed to higher Hg levels compared to females. In this study, 71.3% of males and 49% of females showed blood Hg levels of ≥2.75 μg/L; therefore, the effects of Hg may have been obscured in females.

It is noteworthy that levels of Pb and Cd above the population averages were not significantly related to dyslipidemia despite the ability of these heavy metals to alter lipid metabolism. Indeed, chronic oral exposure to heavy metals, including Pb and Cd, affected the oxidative stress level and did not alter lipid profiles with oxidative stress in a rat model [33]. In this study, although not statistically significant, Cd exposure showed a tendency, especially for males with a prevalence of dyslipidemia. Cd can affect human biological systems at very low doses [34]. In this study, we compared people who were exposed to Cd at higher-than-average levels, which might have obscured the effects of Cd on dyslipidemia. Therefore, the association between Cd and dyslipidemia cannot be completely ruled out.

Dyslipidemia is one of the most well-known risk factors for cardiovascular disease, which is the leading cause of death worldwide. This means that the mortality rate associated with cardiovascular disease could be lowered by identifying the factors related to dyslipidemia and the implementation of comprehensive management [35]. In particular, various factors, including sex, are associated with dyslipidemia [36]. The present study found that age was significantly associated with dyslipidemia in both males and females. Factors including household income and BMI also showed significant associations with dyslipidemia. These relationships are speculated to be due to dietary habits playing an important role in modulating the blood lipid profile. A previous study found that the diet quality differed significantly according to age, sex, and household income in Americans [37], and that subjective health status was associated with dyslipidemia in both sexes. It is therefore necessary to consider these related factors when developing interventions for dyslipidemia.

This study was subject to several limitations. It was not possible to determine the causal relationships between the variables due to the cross-sectional design used to analyze the secondary data. Therefore, we sought to minimize this influence by considering confounding variables, including sociodemographic factors, health behaviors, and mental health factors. Nevertheless, considering that dyslipidemia is a complex metabolic disease involving various risk factors, similar replication studies should continue to be carried out to ensure that there is an association between dyslipidemia and heavy metals. In addition, we could not analyze biomarkers of oxidative stress that are suspected to underlie the effects of Hg on lipid metabolism. Thus, further studies are warranted to identify the underlying mechanisms using a variable for measuring oxidative stress.

## 5. Conclusions

In adult males, exposure to Hg above the average level for the total population is positively associated with dyslipidemia, while such an association was not found in females. These results provide the basis for targeted prevention strategies against dyslipidemia using lifestyle guidelines for reducing Hg exposure and healthy behavioral interventions. However, further studies are needed to reveal causal relationships and to identify the mechanisms underlying this interrelation at the genetic, epigenetic, and biochemical levels.

## Figures and Tables

**Table 1:** General characteristics of the study participants.

Variables		Items	Total Sample		Male		Female	
			Unweighted No.	Weighted% (SE)	Unweighted No.	Weighted% (SE)	Unweighted No.	Weighted% (SE)
Socio-demographic factors	Age (years) ^+^		5345	43.7 (0.3)	2424	42.8 (0.4)	2921	44.6 (0.4)
	Marital status	Yes	3997	69.1 (0.8)	1696	64.9 (1.1)	2301	73.2 (1.0)
		No	1348	30.9 (0.8)	728	35.1 (1.1)	620	26.8 (1.0)
	Education	≤Elementary	1189	18.2 (0.6)	444	13.9 (0.7)	745	22.4 (1.0)
		Middle school	633	12.4 (0.6)	301	12.6 (0.8)	332	12.3 (0.8)
		High school	1529	32.6 (0.9)	696	33.3 (1.2)	833	32.0 (1.1)
		≥University	1744	36.7 (0.9)	857	40.1 (1.3)	887	33.3 (1.0)
	Occupation	WC worker	1174	26.4 (0.8)	603	30.3 (1.2)	571	22.6 (0.9)
		PC worker	591	13.0 (0.6)	216	11.5 (0.8)	375	14.4 (0.9)
		BC worker	494	11.3 (0.6)	416	19.7 (1.2)	78	3.1 (0.4)
		AL worker	618	10.9 (0.6)	272	10.4 (0.7)	346	11.4 (0.8)
		Unemployed	1930	38.3 (0.9)	634	28.0 (1.2)	1296	48.4 (1.1)
	Household income	Lowest	906	13.8 (0.7)	369	12.4 (0.9)	537	15.3 (0.9)
		Lower middle	1315	23.1 (0.9)	589	22.6 (1.1)	726	23.7 (1.1)
		Upper middle	1495	30.0 (1.0)	716	31.6 (1.2)	779	28.4 (1.2)
		Highest	1614	33.0 (1.2)	740	33.5 (1.5)	874	32.6 (1.4)
	Residential area	Urban	4382	86.5 (1.6)	1990	86.7 (1.6)	2392	86.2 (1.6)
		Rural	963	13.5 (1.6)	434	13.3 (1.6)	529	13.8 (1.6)
Health behaviors	Drinking	No	907	15.6 (0.6)	302	11.5 (0.8)	605	19.8 (0.9)
		Yes	4368	84.4 (0.6)	2088	88.5 (0.8)	2280	80.2 (0.9)
	Smoking	No	2853	57.6 (0.8)	486	25.1 (1.2)	2367	89.7 (0.7)
		Yes	1906	42.4 (0.8)	1628	74.9 (1.2)	278	10.3 (0.7)
	BMI	Normal	3216	60.3 (0.9)	1380	55.8 (1.2)	1836	64.9 (1.1)
		Overweight	1502	27.7 (0.8)	813	34.1 (1.1)	689	21.3 (0.9)
		Obesity	279	5.4 (0.4)	105	5.1 (0.5)	174	5.6 (0.5)
		Underweight	341	6.6 (0.4)	124	5.0 (0.5)	217	8.2 (0.7)
	Dyslipidemia	No	3988	84.1 (0.6)	1818	85.9 (0.8)	2170	82.4 (0.9)
		Yes	841	15.9 (0.6)	330	14.1 (0.8)	511	17.6 (0.9)
Mental health	Subjective health status	Good	1610	33.0 (0.8)	826	36.9 (1.1)	784	29.1 (1.1)
		Moderate	2628	51.5 (0.8)	1138	49.3 (1.2)	1490	53.7 (1.2)
		Poor	881	15.5 (0.7)	346	13.8 (0.9)	535	17.1 (0.9)
	Stress level	Low	789	14.2 (0.6)	383	15.4 (0.8)	406	13.1 (0.7)
		Moderate	2909	56.7 (0.8)	1317	56.3 (1.2)	1592	57.0 (1.1)
		High	1455	29.1 (0.8)	624	28.3 (1.1)	831	29.9 (1.0)
Heavy metal exposure	Pb	<1.60 μg/dL	2681	53.6 (1.0)	910	42.1 (1.3)	1771	65.0 (1.1)
		≥1.60 μg/dL	2664	46.4 (1.0)	1514	57.9 (1.3)	1150	35.0 (1.1)
	Hg	<2.75 μg/L	2407	44.7 (1.0)	839	34.6 (1.2)	1568	54.8 (1.4)
		≥2.75 μg/L	2938	55.3 (1.0)	1585	65.4 (1.2)	1353	45.2 (1.4)
	Cd	<0.36 μg/L	689	15.9 (0.7)	403	19.4 (1.1)	286	12.5 (0.8)
		≥0.36 μg/L	4656	84.1 (0.7)	2021	80.6 (1.1)	2635	87.5 (0.8)

Note: WC—white-collar, PC—pink-collar, BC—blue-collar, AL—agribusiness and low-level, BMI—body mass index, Pb—lead, Hg—mercury, Cd—cadmium; + weighted mean with SE.

**Table 2:** Weighted prevalence of dyslipidemia according to sex.

Characteristics		Total Sample			Male			Female		
		Non-Dyslipidemia	Dyslipidemia	*p*-Value	Non-Dyslipidemia	Dyslipidemia	*p*-Value	Non-Dyslipidemia	Dyslipidemia	*p*-Value
		Weighted% (SE)			Weighted% (SE)			Weighted% (SE)		
Age (years) ^+^		44.56 (0.33)	59.90 (0.50)	<0.001	44.45 (0.41)	57.18 (0.77)	<0.001	44.68 (0.39)	62.06 (0.57)	<0.001
Marital status	Yes	80.3 (0.8)	19.7 (0.8)	<0.001	82.0 (1.1)	18.0 (1.1)	<0.001	78.8 (1.1)	21.2 (1.1)	<0.001
	No	96.9 (0.6)	3.1 (0.6)		96.4 (0.9)	3.6 (0.9)		97.7 (0.9)	2.3 (0.9)	
Education	≤Elementary school	64.2 (2.1)	35.8 (2.1)	<0.001	74.9 (3.4)	25.1 (3.4)	<0.001	59.4 (2.6)	40.6 (2.6)	<0.001
	Middle school	74.5 (2.3)	25.5 (2.3)		81.8 (3.3)	18.2 (3.3)		67.4 (3.5)	32.6 (3.5)	
	High school	86.8 (1.0)	13.2 (1.0)		88.1 (1.3)	11.9 (1.3)		85.5 (1.5)	14.5 (1.5)	
	≥University	90.3 (0.8)	9.7 (0.8)		87.2 (1.3)	12.8 (1.3)		94.0 (0.9)	6.0 (0.9)	
Occupation	WC worker	90.4 (1.0)	9.6 (1.0)	<0.001	87.6 (1.5)	12.4 (1.5)	0.550	94.1 (1.1)	5.9 (1.1)	<0.001
	PC worker	85.6 (1.6)	14.4 (1.6)		86.4 (2.6)	13.6 (2.6)		85.0 (2.1)	15.0 (2.1)	
	BC worker	85.2 (1.8)	14.8 (1.8)		85.8 (1.8)	14.2 (1.8)		82.0 (5.4)	18.0 (5.4)	
	AL worker	79.5 (1.9)	20.5 (1.9)		86.0 (2.5)	14.0 (2.5)		73.7 (2.7)	26.3 (2.7)	
	Unemployed	79.3 (1.1)	20.7 (1.1)		83.7 (1.8)	16.3 (1.8)		77.0 (1.5)	23.0 (1.5)	
Household income	Lowest	78.4 (1.8)	21.6 (1.8)	<0.001	83.3 (2.3)	16.7 (2.3)	0.654	74.6 (2.6)	25.4 (2.6)	<0.001
	Lower middle	82.8 (1.3)	17.2 (1.3)		85.9 (1.7)	14.1 (1.7)		79.9 (1.8)	20.1 (1.8)	
	Upper middle	84.7 (1.2)	15.3 (1.2)		86.8 (1.6)	13.2 (1.6)		82.4 (1.7)	17.6 (1.7)	
	Highest	87.2 (1.0)	12.8 (1.0)		86.3 (1.5)	13.7 (1.5)		88.0 (1.3)	12.0 (1.3)	
Residential area	Urban	84.0 (0.7)	16.0 (0.7)	0.597	85.4 (0.9)	14.6 (0.9)	0.182	82.6 (1.0)	17.4 (1.0)	0.574
	Rural	84.8 (1.4)	15.2 (1.4)		88.6 (2.0)	11.4 (2.0)		81.2 (2.3)	18.8 (2.3)	
Drinking	No	73.0 (2.4)	27.0 (2.4)	<0.001	84.9 (3.3)	15.1 (3.3)	0.732	69.2 (2.8)	30.8 (2.8)	<0.001
	Yes	85.2 (0.7)	14.8 (0.7)		86.0 (0.9)	14.0 (0.9)		84.2 (1.0)	15.8 (1.0)	
Smoking	No	83.4 (0.8)	16.6 (0.8)	0.195	90.6 (1.5)	9.4 (1.5)	0.002	81.5 (1.0)	18.5 (1.0)	0.014
	Yes	84.9 (0.9)	15.1 (0.9)		84.4 (1.0)	15.6 (1.0)		88.6 (2.3)	11.4 (2.3)	
BMI	Normal	86.3 (0.8)	13.7 (0.8)	<0.001	87.1 (1.1)	12.9 (1.1)	0.042	85.6 (1.0)	14.4 (1.0)	<0.001
	Overweight	79.2 (1.2)	20.8 (1.2)		83.8 (1.4)	16.2 (1.4)		71.8 (2.1)	28.2 (2.1)	
	Obesity	77.3 (2.8)	22.7 (2.8)		82.7 (4.0)	17.3 (4.0)		72.4 (4.0)	27.6 (4.0)	
	Underweight	97.3 (1.2)	2.7 (1.2)		96.0 (2.8)	4.0 (2.8)		98.0 (1.1)	2.0 (1.1)	
Subjective health status	Good	91.3 (0.9)	8.7 (0.9)	<0.001	91.2 (1.2)	8.8 (1.2)	<0.001	91.4 (1.3)	8.6 (1.3)	<0.001
	Moderate	84.2 (0.9)	15.8 (0.9)		86.7 (1.2)	13.3 (1.2)		81.9 (1.2)	18.1 (1.2)	
	Poor	70.8 (2.0)	29.2 (2.0)		72.0 (2.9)	28.0 (2.9)		69.9 (2.4)	30.1 (2.4)	
Stress level	Low	83.5 (1.5)	16.5 (1.5)	0.191	85.9 (2.2)	14.1 (2.2)	0.292	80.9 (2.1)	19.1 (2.1)	0.569
	Moderate	83.4 (0.8)	16.6 (0.8)		84.9 (1.2)	15.1 (1.2)		81.9 (1.2)	18.1 (1.2)	
	High	85.7 (1.1)	14.3 (1.1)		88.1 (1.5)	11.9 (1.5)		83.5 (1.7)	16.5 (1.7)	
Pb exposure	<1.60 μg/dL	87.5 (0.8)	12.5 (0.8)	<0.001	89.1 (1.2)	10.9 (1.2)	0.003	86.5 (1.1)	13.5 (1.1)	<0.001
	≥1.60 μg/dL	80.9 (1.0)	19.1 (1.0)		84.0 (1.1)	16.0 (1.1)		75.7 (1.7)	24.3 (1.7)	
Hg exposure	<2.75 μg/L	86.4 (0.9)	13.6 (0.9)	0.003	90.5 (1.3)	9.5 (1.3)	<0.001	84.1 (1.2)	15.9 (1.2)	0.046
	≥2.75 μg/L	82.6 (0.9)	17.4 (0.9)		84.0 (1.1)	16.0 (1.1)		80.5 (1.4)	19.5 (1.4)	
Cd exposure	<0.36 μg/L	97.3 (0.8)	2.7 (0.8)	<0.001	97.4 (1.0)	2.6 (1.0)	<0.001	97.3 (1.2)	2.7 (1.2)	<0.001
	≥0.36 μg/L	82.7 (0.7)	17.3 (0.7)		84.2 (0.9)	15.8 (0.9)		81.4 (1.0)	18.6 (1.0)	

Note: WC—white-collar, PC—pink-collar, BC—blue-collar, AL—agribusiness and low-level, BMI—body mass index, Pb—lead, Hg—mercury, Cd—cadmium; ^+^ weighted mean with SE.

**Table 3:** Factors affecting dyslipidemia risk according to sex.

Characteristics		Total Sample		Male		Female	
		OR	95% CI	OR	95% CI	OR	95% CI
Age		**1.068** ***	1.056–1.080	**1.066** ***	1.049–1.084	**1.079** ***	1.062–1.097
Marital status	Yes	1		1		1	
	No	0.748	0.456–1.229	0.846	0.457–1.563	0.802	0.318–2.027
Education	≤Elementary	1		1		1	
	Middle school	0.971	0.704–1.340	0.934	0.525–1.660	1.162	0.763–1.769
	High school	0.900	0.662–1.224	1.124	0.691–1.831	0.923	0.604–1.411
	≥University	0.773	0.538–1.110	1.244	0.694–2.230	0.620	0.358–1.072
Occupation	WC worker	1		1		1	
	PC worker	0.989	0.690–1.419	1.226	0.706–2.130	0.855	0.501–1.457
	BC worker	0.855	0.566–1.291	0.960	0.568–1.625	0.996	0.468–2.119
	AL worker	0.800	0.548–1.167	0.577	0.297–1.119	1.066	0.608–1.867
	Unemployed	0.878	0.643–1.198	0.689	0.409–1.160	1.008	0.606–1.676
Household income	Lowest	1		1		1	
	Lower middle	**1.571** *	1.085–2.275	1.210	0.679–2.156	**1.980** **	1.243–3.152
	Upper middle	**1.872** **	1.283–2.732	1.307	0.730–2.340	**2.376** ***	1.502–3.757
	Highest	**1.621** *	1.122–2.342	1.257	0.710–2.223	**1.744** *	1.094–2.782
Drinking	No	1		1		1	
	Yes	1.032	0.758–1.405	1.109	0.582–2.117	1.125	0.773–1.636
Smoking	No	1		1		1	
	Yes	0.889	0.714–1.107	1.101	0.699–1.735	0.958	0.558–1.645
BMI	Normal	1		1		1	
	Overweight	**1.558** ***	1.261–1.924	**1.423** *	1.036–1.956	**1.567** **	1.163–2.111
	Obesity	**2.184** ***	1.454–3.282	**2.016** *	1.043–3.896	**2.078** **	1.258–3.433
	Underweight	**0.192** **	0.070–0.533	**0.183** *	0.036–0.940	**0.221** *	0.066–0.747
Subjective health status	Good	1		1		1	
	Moderate	**1.821** ***	1.392–2.381	1.412	0.942–2.116	**2.141** ***	1.422–3.225
	Poor	**3.115** ***	2.281–4.252	**3.621** ***	2.313–5.668	**2.665** ***	1.688–4.209
Pb exposure	<1.60 μg/dL	1		1		1	
	≥1.60 μg/dL	0.882	0.699–1.112	0.774	0.544–1.101	0.956	0.708–1.291
Hg exposure	<2.75 μg/L	1		1		1	
	≥2.75 μg/L	**1.273** *	1.025–1.580	**1.699** **	1.150–2.511	1.131	0.863–1.482
Cd exposure	<0.36 μg/L	1		1		1	
	≥0.36 μg/L	1.724	0.940–3.163	2.033	0.877–4.712	1.051	0.387–2.853

Note: Bolded numbers represent statistically significant values; WC—white-collar, PC—pink-collar, BC—blue-collar, AL—agribusiness and low-level, BMI—body mass index, Pb—lead, Hg—mercury, Cd—cadmium; * *p* < 0.05, ** *p* < 0.01, *** *p* < 0.001.

## Data Availability

Not applicable.
